# Presence of Parabens in Different Children Biological Matrices and Its Relationship with Body Mass Index

**DOI:** 10.3390/nu15051154

**Published:** 2023-02-24

**Authors:** Inmaculada Moscoso-Ruiz, Yolanda Gálvez-Ontiveros, Cristina Samaniego-Sánchez, Vega Almazán Fernández de Bobadilla, Celia Monteagudo, Alberto Zafra-Gómez, Ana Rivas

**Affiliations:** 1 Department of Analytical Chemistry, University of Granada, 18071 Granada, Spain; 2 Department of Nutrition and Food Science, University of Granada, 18071 Granada, Spain; 3 Instituto de Investigación Biosanitaria (ibs.GRANADA), 18012 Granada, Spain; 4 Institute of Nutrition and Food Technology (INYTA) “José Mataix Verdú”, Biomedical Research Centre (CIBM), University of Granada, 18100 Granada, Spain; 5 Maracena Healthcare Centre, Camino de los Eriales s/n, 18200 Granada, Spain

**Keywords:** parabens, obesogens, children, urine, nail, saliva

## Abstract

Parabens have been accepted almost worldwide as preservatives by the cosmetic, food, and pharmaceutical industries. Since epidemiological evidence of the obesogenic activity of parabens is weak, the aim of this study was to investigate the association between parabens exposure and childhood obesity. Four parabens (methylparaben/MetPB, ethylparaben/EthPB, propylparaben/PropPB, and butylparaben/ButPB) were measured in 160 children’s bodies between 6 and 12 years of age. Parabens measurements were performed with ultrahigh-performance liquid chromatography coupled with tandem mass spectrometry (UHPLC-MS/MS). Logistic regression was used to evaluate risk factors for elevated body weight associated with paraben exposure. No significant relation was detected between children’s body weight and the presence of parabens in the samples. This study confirmed the omnipresence of parabens in children’s bodies. Our results could be a basis for future research about the effect of parabens on childhood body weight using nails as a biomarker due to the ease of its collection and its non-invasive character.

## 1. Introduction

Overweight and obesity are defined as abnormal or excessive fat accumulation that may cause health disorders such as cardiovascular diseases, musculoskeletal conditions, alterations in spinal posture and mobility, metabolic syndrome, and gastrointestinal and pulmonary conditions [1,2]. Recent data from World Health Organization showed that 39 million children under the age of 5 were overweight or obese in 2020, and nearly tripled since 1975 [3]. Overweight/obesity can be attributed to several factors, but traditional risk factors such as diet, physical activity, and genetics cannot completely explain the increase [4]. In 2006 Grün and Blumberg postulated the obesogens hypothesis, where certain environmental pollutants could induce adipogenesis [5]. Some endocrine-disrupting chemicals (EDCs) have been catalogued as obesogens due to the effect they induce in adipose tissue deposition via mimicking endogenous endocrine hormones or activating lipidogenesis-related nuclear receptors [6].

Parabens are EDCs widely used as preservatives in the cosmetic, pharmaceutical, and food industries because of their antimicrobial properties and low allergenic potential [7,8,9]. Additionally, their chemical stability, low toxicity, and allergenicity lead parabens to almost worldwide acceptance [7,8,9,10]. The most commonly used parabens are methylparaben (MetPB), ethylparaben (EthPB), propylparaben (PropPB) and butylparaben (ButPB) [11]. All four of these parabens are allowed in personal care products (PCPs), yet both PropPB and ButPB are prohibited in food products due to scientific evidence of their toxicity [12]. The main entry routes of parabens to the body are dermal (by cosmetics), oral (by foods and medicines), and by inhalation [9,13].

It has been shown that parabens may modulate or alter the endocrine system and, therefore, may have negative effects on health due to their cumulative nature in tissues [11,14,15]. In addition, the obesogenic effects of parabens have recently been demonstrated [16,17]. Fat accumulation in the body may occur by increasing the number of adipocytes and their volume or altering the pathways of adipocyte tissue control. Obesogens such as parabens can act by modifying the number or volume of adipocytes by interfering with transcriptional regulators that control lipid flux, adipocyte proliferation, and adipocyte differentiation, particularly through the peroxisome proliferator (PPAR) [18]. In addition, parabens have been shown to promote adipocyte differentiation in 3T3-L1 cells, whose action increased in conjunction with the linear length of the alkyl chain associated with PPAR γ activation [17].

Available literature has shown the obesogenic effect of parabens in vitro and in vivo, yet there still is no consensus on their obesogen effect on the human body [19]. Recently we demonstrated an association between dietary exposure to parabens and overweight/obesity in Spanish adolescent girls using dietary records [16]. However, the use of biological matrices could give us more detailed information on the subjects’ exposure to obesogens.

Urine has been the traditional matrix of choice to study paraben concentrations in the human body. Several studies have analysed the possible relationship between parabens in urine and overweight/obesity in different populations of both children [20,21] and adults [22,23,24], finding a direct statistically significant relationship between MetPB, EthPB, and PropPB and obesity. However, other authors do not confirm those results [25,26,27,28]. The majority of the studies were carried out in Asia, and only one was carried out in a Spanish child population with an average age of 11, showing non-statistically significant results [29]. Children are particularly vulnerable to obesogens because of the critical stage they’re in for the correct functioning and development of the endocrine system [30].

Since urine only provides information on fairly recent exposures, other matrices should be studied as bioindicators of body contamination. Blood might be a good matrix if it were not so invasive. Several studies have considered other non-invasive matrices to study the presence of parabens, such as saliva [31] or nail [32]. The nail can provide information on bioaccumulation over time. On the other hand, Barbosa et al. have made comparisons between the concentration of some contaminants in blood and saliva [33].

Due to the lack of consensus as well as the ubiquity of parabens in foods and cosmetics [13,34,35,36], it is necessary to study alternative matrices to better chart their bioaccumulation in the body, as well as examine more population groups to better understand their obesogen effects, especially in vulnerable populations such as children. The present work aims to study the presence of parabens in a range of biological matrices (nails, urine, and saliva) in the child population of Granada (Spain), as well as the possible association of these parabens with overweight and/or obesity.

## 2. Materials and Methods

### 2.1. Study Design and Settings

The present research is a case-control study designed to assess the influence of environmental factors on the development of overweight and obesity among Spanish children and adolescents. Study participants were recruited from January 2020 to January 2022 from different primary care centres and schools in the province of Granada, Spain. Protocol was approved by the Ethics Committee of Provincial Biomedical Research of Granada (CEI). All parents or legal tutors of study participants were fully informed about the study objectives, and they signed informed consent. Personal identifiers in the dataset were removed to guarantee data confidentiality.

### 2.2. Study Participants

Eligible cases met the following inclusion criteria: (1) diagnosis of overweight or obesity; (2) children aged 6 to 12 years old; (3) having resided within the study area continuously for at least 6 months. The controls had to meet the same inclusion criteria, except for the diagnosis of overweight and obesity. Individuals with obesity associated with a pathology or pharmacological treatment were excluded from the study. The sample included a total of 231 subjects.

For this study, the aim of which was to investigate the relationship between paraben exposure and obesity, only 160 participants (53.5% boys) of the 231 who agreed to participate were included and displayed concentrations of these compounds in urine, saliva and/or nails (in one, two or the three matrices). No statistically significant differences between controls and cases included/not included were observed, except for the marital status of parent’s cases among controls (Supplementary Table S1).

### 2.3. Data Collection

To achieve the objective of this study, data were collected and used on variables related to anthropometry (weight and height), sociodemographics (gender, age, occupational rank, and marital status of parents or legal guardians), lifestyle (energy intake, physical activity, and smoking habits), urine creatinine levels and detected levels of parabens in the three biological matrices.

Height data were obtained with a tallimeter (model SECA 214 (20–207 cm) and weight with a portable Tanita floor scale (model MC 780-S MA). Body mass index (BMI) was calculated as weight in kilograms divided by height squared in meters. The sample population was classified as underweight, normal weight, and overweight or obese, as described by Cole et al. (2000, 2007) [37,38]. According to this classification, we divided our population into two groups: cases which included overweight and obese children, and controls which included normal-weight children. The cut-off point was established in the equivalent value of 25 kg m^−2^ for adults.

Data on sociodemographic variables and lifestyle were obtained after completing a questionnaire supervised by a nutritionist. The occupational rank of the parents was determined based on the occupational classifications of the international standard [39]. Energy intake was obtained from three 24 h recalls. The questionnaire was addressed to the parents or legal guardians of the population and completed by nutrition professionals.

Urine creatinine levels were analysed by the Ángel Mendez Soto Clinical Analysis Laboratory. The classical Jaffé method [40,41] based on the photometric measurement of the kinetics of creatinine reaction with picric acid at 37 °C was employed. A kit of reagents was provided by Biosystems (Barcelona, Spain).

### 2.4. Determination of Parabens in Biological Samples

A total of 4 parabens (MetPB, EthPB, PropPB, and ButPB) were determined in nails, saliva, and urine samples. Saliva and urine samples were stored at −80 °C until laboratory treatment; nails were stored at room temperature. All samples were taken between 1 and 4 months since the questionnaire was completed. Parameters of validation, LOD, LOQ, recovery, calibration range, etc., can be checked in previously published works of our group research [31,32,42]. For paraben determinations in biological samples, three replicas/samples were analysed and the relative standard deviation was <15% in all cases.

#### 2.4.1. Determination of Parabens in Saliva

Saliva (*n* = 89) was collected in wide-mouthed glass jars. The collection technique was performed by passively collecting saliva in the mouth and prior fasting by the subjects (overnight). Passive drool collection from children was carried out over several weekends until at least 4 g of sample was collected.

The methodology followed for the extraction is described in the article Moscoso-Ruiz et al., 2022 [31]. Briefly, for saliva treatment, 1 g of each saliva sample was weighed into a 10 mL glass tube to which 2 mL of acetonitrile was added. Additionally, 150 µL of acetic acid solution (0.1 M) was added. The mixture was vortexed for 30 s and centrifuged for 5 min. The supernatant was transferred to a 10 mL glass tube and evaporated to dryness. A volume of 1.5 mL of acetone as extraction solvent was added to the resulting residue, and ultrasound-assisted extraction was performed for 30 min at 35% power. The mixture was centrifuged, and the supernatant was transferred to another 10 mL tube. The extraction was then repeated with 1.5 mL of ethanol (EtOH) using the same conditions. The supernatant was evaporated to dryness, reconstituted with 20 µL of methanol (MeOH) and 80 µL of ultrapure water, and then centrifuged. It was analysed with ultra-high-performance liquid chromatography coupled with a triple quadrupole tandem mass spectrometry (UHPLC-MS/MS) system.

#### 2.4.2. Determination of Parabens in Urine

Polypropylene beakers were previously analysed to ensure they did not contain parabens and were used for urine collection (*n* = 149). Urine was collected first in the morning, prior to fasting subjects.

The methodology for the determination of total parabens in the urine samples followed the procedure outlined by Moscoso-Ruiz et al., 2022 [42]. First, enzymatic treatment was carried out by adding 25 µL of β-glucuronidase/sulfatase and 100 µL of β-glucuronidase to a 4 mL of sample to know the total content in parabens. Incubation time was 24 h at 37 °C. After that, it was added 4 mL of a 10% (*w*/*v*) of sodium chloride aqueous solution and 100 μL of hydrochloric acid (6 N) until pH 2 was reached. Then dispersive liquid-liquid microextraction was performed. A mixture of 400 μL of acetone and 600 μL of chloroform was rapidly injected into the urine sample with a Hamilton syringe. Subsequently, the samples were gently vortexed and centrifuged for 5 min. The low phase was collected and transferred to another 10 mL glass tube. The extraction was repeated 4 times in total, and the organic phase was evaporated to dryness. The solid residue was reconstituted with 20 µL of MeOH and 80 µL of ultrapure water and, following centrifugation, was injected into the UHPLC-MS/MS system.

#### 2.4.3. Determination of Parabens in Nails

Fingernails and toenails were collected over a 3-month period (*n* = 74). For the extraction and determination of parabens in nails, the methodology described by Martín-Pozo et al., 2020 [32] was followed. Individual nails were first cleaned, as per Martín-Pozo et al., 2020 to remove any external contamination [32]. Both fingers and toenails were lyophilized and crushed in a ball mill until the powder was obtained. Aliquots of 0.1 g of lyophilized nails were prepared with 1 mL of sodium hydroxide/MeOH (0.04 mol/L) and incubated at 30 °C for 15 h. The mixture was previously vortexed for 2 min. After incubation, the digested nails were cooled to room temperature and, following centrifugation for 10 min, were evaporated to dryness under a stream of nitrogen gas. The residue was reconstituted with 20 µL of MeOH and 80 µL of ultrapure water, centrifuged, and analysed in the UHPLC-MS/MS system

### 2.5. Statistical Analysis

Continuous and parametric variables (height and energy intake) were described as mean and standard deviation (SD), while continuous and non-parametric variables (weight, urinary creatinine levels, and paraben concentration in biological samples) were described as the median and interquartile range (IQR). Absolute and relative frequency distributions were calculated for categorical variables (gender, age, physical activity, smoking habits, educational level, and marital status of parents or legal guardians).

The Student’s *t*-test, Mann-Whitney U test, and Pearson’s chi-square test were used to assess the differences between cases and controls for parametric, non-parametric, and categorical variables, respectively.

A logistic regression analysis was used to determine the association between overweight and obesity (cut-off point equivalent to 25 kg m^−2^ in adults, according to Cole et al. [37,38]) as a dependent variable, and concentrations of parabens (ng g^−1^ or ng mL^−1^) in the three biological samples (nails, urine, and saliva) as independent variables. For all analyses performed, independent variables were dichotomized according to the median value (reference category: concentration ≤ median value) or according to [limit of detection (LOD)/√2] values for the analytes that were not detected in more than 30% of samples [43]. Odds ratio (OR) and 95% confidence intervals (CI) were calculated for crude and adjusted models. For the crude model, independent variables were included one by one separately, while for the adjusted model, these variables were included together with confounder factors using the ented method. Energy intake, physical activity, parents’ level of education, smoking habits, and marital status were all considered as confounder factors in the adjusted model when they modified the OR value by over 10% in crude analysis. Regarding creatinine in urine samples, it was decided to use creatinine levels as an independent variable in all regression models rather than urine standardization. This approach is less likely to produce a skewed estimate of the effect [44]. Given that ButPB was detected in a very low percentage of samples analysed, this contaminant was not included in the logistic regression analysis.

SPSS v.23 (version 23, IBM^®^ SPSS^®^ Statistics, Armonk, NY, USA) was used for all statistical analyses; significance was set at *p* < 0.05.

## 3. Results

Characteristics of the study population are shown in Table 1. Weight and height were significantly higher in the case group (*p* < 0.001), while the parents’ level of education was significantly lower (*p* < 0.001). More children with married parents were found in the control group and more divorcee parents in the case group (*p* = 0.010). Regarding gender, age, energy intake, physical activity, and smoking habits, non-significant differences were observed between cases and controls.

After determining paraben concentrations in the three biological samples (Table 2), nails were found to be the matrix with the highest total paraben values (*p* < 0.001), EthPB (*p* < 0.001), PropPB (*p* < 0.001) and ButPB (*p* < 0.001) (data not showed). EthPB was higher in controls, but non-significant differences were observed with respect to the cases. PropPB and total parabens in urine were significantly higher among cases. Analytic methods used to determine parabens in nails did not show results for MetPB due to the high concentration of this compound in this matrix.

Table 3, Table 4 and Table 5 show the influence of exposure to MethPB, EthPB, and PropPB, and the total concentration of Parabens as determined by paraben presence in nails, urine, and saliva, respectively, on the overweight and obesity of the study population. The association of all of them (individually and in summation) with overweight and obesity was not significant. After adjusting for gender, age, creatinine level (for urine), and other confounding factors, OR values for PropPB, paraben totals in nails (Table 3), and all analytes, including total parabens in urine (Table 4) showed that for those values which were lower than median or LOD values, the study population showed lower body weight. However, this relationship was not significant.

## 4. Discussion

The objective of this work was to analyse the association between paraben concentrations in three biological matrices (nails, urine, and saliva) and overweight/obesity in children. The results found at least one of the four studied parabens in each biological sample. Data showed that those children with a BMI ≥ 25 kg m^−2^ have higher paraben concentrations in biological samples versus the control group (BMI < 25 kg m^−2^), although these findings are not statistically significant.

The effect of parabens on obesity has been analysed in different in vitro, in vivo, and epidemiological studies [22,45,46]. In vitro studies analysed the influence of parabens on adipocyte differentiation. Hu et al. (2013) [45] studied the effects of parabens on adipocyte differentiation, finding that the distinction is higher when the linear alkyl chain increases, with ButPB being the compound with more potency to modulate and promote the early phases of the differentiation. Additionally, the study tested the transactivation of the glucocorticoid receptor and/or the peroxisome proliferator-activated receptor (PPARγ) by parabens, obtaining an increment of potency when the radical chain is larger [45]. Both mechanisms are established as signalling pathways in adipocyte differentiation. Another study suggests that parabens may modulate stem cell fate by favouring the differentiation of adipocytes at the expense of osteoblast or chondrocytes since these cell types are known to share the same stem cell population [47]. In vivo studies directly analysed the influence of one or more parabens in a living organism. Boberg et al. (2008) [48] evidenced in rat foetuses that ButPB reduced levels of leptin, a hormone involved in body weight regulation. On the other hand, Leppert et al. (2020) [46] demonstrated in an in vivo mouse model that maternal exposure to ButPB increases food intake and weight gain in female offspring by neuronal dysregulation of satiety, involving the appetite-regulating gene Proopiomelanocortin.

Most of the epidemiological studies that analysed the effect of parabens on BMI used urine as a matrix. Kang et al. (2016) [22] studied the urine of 2541 Korean individuals aged 3 to 69 years old, finding that MetPB and PropPB concentrations were correlated in this matrix and were positively associated with BMI [22]. Li et al. (2019) [23] also found a moderate correlation between PropPB and BMI in the urine of pregnant women with gestational diabetes mellitus [23]. In 2020, another study in Canada used a population of 2564 children and adults aged 3 to 79 years old to evaluate, among other things, the relationship between the presence of parabens in urine and BMI and metabolic syndrome. As opposed to the previous study, Kim and Chevrier, 2020 found an inverse association between EthPB and BMI in women, and there were no associations in children’s populations [27]. However, the results of this investigation showed that EthPB in children’s urine had higher OR compared with the rest of the PBs and lower *p*, though still over 0.05. Lee et al. (2021) [24] found the same positive urinary correlation trend between EthPB and BMI in a population of 3779 adults aged 19 to 86 years old. Feizabadi et al. (2020) [26] studied parabens in 178 urine samples of Iranian adults, also finding a correlation between MetPB and PropPB levels; however, they did not find a correlation between this family of chemicals and BMI or waist circumference, except for MetPB displaying an inverse correlation [26]. Yet another study showed that urinary levels of parabens and BMI have no correlation whatsoever [49], the study population being 52 young Indian women between 18 and 31 years old. Another two studies obtained inverse correlations between parabens in urine and BMI [28,50]. Xu et al. (2022) [28] studied several EDCs in the urine of 300 Chinese people aged 2 to 80 years old, and Hajizadeh et al. (2020) [50] evaluated 95 Iranian pregnant women as the study population. Results from both papers showed that EthPB and BMI were inversely proportional to each other, while Hajizadeh et al. (2020) [50] found a possible relation between EthPB and PropPB levels.

To the best of our knowledge, only a few articles have studied the relationship between BMI and levels of PBs in children, using urine as a matrix. Berger et al. (2021) [21] studied a population of 309 children aged five using two different statistical models to study several phenols, both showing a correlation between PropPB in urine and BMI [21]. Guo et al. (2017) [20] studied a population of 436 children aged three, finding a direct correlation between levels of EthPB and BMI. Conversely, Quirós-Alcalá et al. (2018) [25] studied a 1324 children and adolescent population aged 6 to 19 years old inverse, finding a correlation between MetPB and BMI [25]. In the USA, Deierlein et al. (2017) [51] did not find a correlation between parabens and BMI in a population of 1017 girls between six and eight years old. Similarly, Güil-Oumrait et al. (2022) [29] did not observe a correlation in a population of 1015 Spanish children with a mean age of 11. The results of this study showed that MetPB and PropPB concentrations in urine did not show any significant statistical correlation with overweight/obesity.

Besides urine, three authors have used alternative biological matrices to analyse the relationship between paraben concentrations in biological samples and body weight. In 2017, van der Meer et al. [52] studied two different regions of the human brain in 24 subjects. Only MetPB was detected, and found no correlation with BMI [52]. Reimann et al. (2021) [53] studied the association between EthPB in the placenta and BMI, concluding that prenatal EthPB exposure may affect early childhood BMI [53]. Artacho-Cordón et al. (2018) [54] directly analysed adipose tissue samples in a population of 144 adults, given that 2% of total parabens remain in body tissues after subcutaneous administration [55]. Results showed that almost 90% of the samples were positive for ≥4 contaminants, MetPB and PropPB being the most prevalent parabens. However, they did not find a statistically significant correlation between these parabens and the weight of the individuals.

In this current research, nails and saliva were used due to the ease and non-invasiveness of their collection. Parabens in nails obtained a higher OR (2.18), but the results were not statistically significant. In saliva, the lower *p*-value corresponded to EthPB in an inverse relationship with overweight/obesity, although still not significant (>0.05). There are no other epidemiological studies involving these biological matrices. Table 6 shows a summary of consulted papers that study the relationship between BMI and the levels of parabens in a range of biological matrices.

To the best of our knowledge, this is the first study simultaneously involving parabens in three different biological matrices and their relationship with overweight/obesity. The available literature is scarce, and the majority of the works just use urine levels to study their correlation with overweight/obesity. Urine is the biological matrix traditionally used due to the ease of its collection and the high quantity of samples that can be obtained in a short period of time. Moos et al. [56] studied the hazard index and daily intake of parabens based on 24 h urinary levels, obtaining that MetPB and EthPB are below levels of health risk, but in 8.4% of the population studied (660 people), levels of PropPB and ButPB were over the hazard intake. However, urine samples have some deficiencies, primarily that the exogenous toxins contained they contain primarily represent the diet of the previous 24/48 h. In the case of the parabens in this research, the larger the radical chain of the paraben, the larger the persistence in the body. A study in 2016 analysed the metabolism and elimination of certain parabens via urine [57]. In the first 24 h, 80% of the paraben dose introduced into the body was recovered; in the first 48 h, almost 85% of MetPB was recovered, while only 80.6% of ButPB was recovered. It can therefore be concluded that PBs bioaccumulate, and hence the information that urine provides us is limited.

In this study, children’s nails and saliva were also analysed. The mouth represents the first step in digestion, being the point of entry for external dietary contamination. Saliva is secreted by the salivary glands, which have high blood flow, and chemicals and their metabolites pass into the saliva via different mechanisms. Saliva has been widely employed for biomonitoring medicines or drugs, although its use for environmental exposure is not yet thoroughly studied [58]. Some studies exist comparing salivary and blood contamination, obtaining comparable results [59]. On the other hand, nails provide information about long-term exposure to contaminants, their collection is easy, and their storage at room temperature is straightforward. Nails have traditionally been used as a biomarker for metals, specifically arsenic [60,61]. However, they have not been explored sufficiently as a biomarker for emerging contaminants, despite their simple composition making them a good matrix to analyse body pollutants.

### Strengths and Weaknesses

This is the first study involving several child biological matrices that explores an association between parabens and body weight. To the best of our knowledge, there are no other epidemiological studies that analyse the relationship between parabens in more than one matrix, as well as with obesity/overweight. Childhood is a critical window of exposure to obesogens, and it has been demonstrated that obese children have more risk of suffering adulthood obesity, not to mention a series of other pathologies related to overweight/obesity. It is, therefore, essential to investigate external factors that promote this health impairment. It is also essential to explore options of matrices, especially non-invasive ones, to better understand the bioaccumulation of these contaminants.

The principal weakness of this study was the sample size. One hundred sixty children participated, and, as per the literature, similar numbers of the individual have been included in studies that analyse the relationship between parabens in biological matrices and obesity [26,49,52,54]. The difficulty of access to children compared to an adult population, exacerbated by the COVID-19 situation, should be taken into account.

## 5. Conclusions

This is the first study to report on paraben concentrations in a range of biological matrices from children and the relationship of these parabens with obesity/overweight. This work explores biological samples to measure the accumulation of obesogens which have not been widely studied yet. Our results show that there are no statistically significant relationships between the presence of parabens in the studied biological samples and body weight. Other epidemiological articles relating PBs to obesity were consulted, and as yet, there is no consensus on the effect of these compounds on body weight. However, as this study confirms, given the omnipresence of parabens in children’s bodies, coupled with increasing childhood obesity worldwide, more investigation is necessary to clarify a possible relationship. These findings could be a basis for future research on the effect of parabens on childhood body weight using nails as a biological matrix due to the ease and non-invasiveness of their collection and the fact that pollutants bioaccumulate in this matrix, thus revealing data on long-term exposure.

## Figures and Tables

**Table 1:** General characteristics of the study population (*n* = 160).

		*n*	Control (*n* = 101)	Cases (*n* = 59)	*p*
Gender (%)	Male	84	58.8	41.2	0.505 ^a^
	Female	76	67.1	32.9	
Age, categorized (%)	6–10 years	125	64.0	36.0	0.280 ^a^
	>10–12 years	35	58.3	41.7	
Weight, kg	Median		25.5	53.3	**<0.001 ^b^**
	IQR		12.6	21.9	
Height, cm	Mean		127.8	140.4	**<0.001 ^c^**
	SD		20.7	12.9	
BMI, kg m^−2^	Mean		16.14	24.45	**<0.001 ^c^**
	SD		0.2	0.5	
Energy Intake, kcal day^−1^	Mean		2011.9	2001.1	0.702 ^c^
	SD		512.4	453.0	
Physical Activity (out-of-school) (%)	No	58	62.1	37.9	0.995 ^a^
	Yes	86	59.3	40.7	
Parents’ Level of Education (%)	Primary	5	20.0	80.0	**<0.001 ^a^**
	Secondary	52	42.3	57.7	
	University	89	74.2	25.8	
Parents’ Smoking Habits (%)	No	126	61.1	38.9	0.740 ^a^
	Yes	32	65.6	34.4	
Parents’ Marital Status (%)	Married	126	65.1	34.9	**0.010 ^a^**
	Divorcee	13	23.1	76.9	
	Single	7	57.1	42.9	
Urinary Creatinine Levels, g L^−1^	Median		0.9	0.9	0.439 ^b^
	IQR		0.6	0.8	

Abbreviations: IQR, Interquartile Range; SD, Standard Deviation. BMI, Body Mass Index. *p*-values < 0.05 are highlighted in bold. ^a^ Chi-square test; ^b^ U Mann-Whitney test; ^c^ Student’s *t*-test.

**Table 2:** Paraben concentration in nails, urine, and saliva (ng g^−1^ or ng mL^−1^).

	**NAILS (ng g^−1^)**								
	**Control (*n* = 52)**					**Cases (*n* = 22)**			
	**% Detection**	**Median**	**P_25_**	**P_75_**	**% Detection**	**Median**	**P_25_**	**P_75_**	p *
MetPB	100.0	>500	>500	>500	100	>500	>500	>500	-
EthPB	98.1	25.70	9.44	77.51	100	15.93	10	169.42	0.692
PropPB	73.1	14.70	<LOD	134.6	68.2	47.85	<LOD	197.97	0.624
ButPB	9.6	<LOD	<LOD	<LOD	13.6	<LOD	<LOD	<LOD	0.622
Paraben total	100.0	47.76	16.47	225.71	100.0	210.91	16.04	1085.19	0.214
	**URINE (ng mL^−1^)**								
	**Control (*n* = 97)**					**Cases (*n* = 52)**			
	**% Detection**	**Median**	**P_25_**	**P_75_**	**% Detection**	**Median**	**P_25_**	**P_75_**	p *
MetPB	91.8	4	2.17	8.93	90.4	4.93	2.63	22.24	0.174
EthPB	26.8	0.21	<LOD	0.13	23.1	0.02	<LOD	<LOD	0.533
PropPB	24.7	0.02	<LOD	0.26	40.4	0.02	<LOD	1.73	**0.044**
ButPB	9.3	<LOD	<LOD	<LOD	5.8	<LOD	<LOD	<LOD	0.468
Paraben total	100.00	4.88	2.37	11.49	98.1	7.24	2.98	27.52	**0.033**
	**SALIVA (ng g^−1^)**								
	**Control (*n* = 58)**					**Cases (*n* = 31)**			
	**% Detection**	**Median**	**P_25_**	**P_75_**	**% Detection**	**Median**	**P_25_**	**P_75_**	p *
MetPB	91.4	0.71	0.71	6.65	87.1	0.71	0.71	8.05	0.904
EthPB	94.8	7.30	3.35	9.45	87.1	6.00	1.15	7.85	0.113
PropPB	87.9	0.71	0.71	1.35	77.4	0.71	0.71	1.1	0.446
ButPB	22.4	<LOD	<LOD	<LOD	22.6	<LOD	<LOD	<LOD	0.943
Paraben total	100.0	11.79	8.53	19.32	100.0	12.23	8.14	16.62	0.993

Abbreviations: LOD, Limit of Detection; P_25_: First quartile; P_75_: Fourth quartile. * U Mann-Whitney test. *p*-values are highlighted in bold. Parabens concentration was calculated by the mean of three replicas with a Relative Standard Deviation (RSD) < 15%.

**Table 3:** Parabens level in nails as influencing factors on overweight/obesity (logistic regression analysis).

	Crude			Adjusted *		
	OR	95% CI	*p*	OR	95% CI	*p*
MetPB (Ref. MetPB concentration ≤ median)	-	-	-	-	-	-
EthPB (Ref. EthPB concentration ≤ median)	0.77	0.28–2.10	0.611	0.77	0.28–2.09	0.601
PropPB (Ref. PropPB concentration ≤ median)	1.69	0.61–4.63	0.311	1.41	0.47–4.21	0.541
Paraben total (Ref. Paraben total concentration ≤ median)	2.21	0.79–6.16	0.131	2.18	0.67–6.96	0.195

Abbreviations: Ref, Reference category; OR, Odds ratio; 95% IC: Confidence interval. * All analytes were adjusted for age and gender. PropPB was also adjusted for energy intake and Paraben total for the level of education.

**Table 4:** Parabens level in urine as influencing factors on overweight/obesity (logistic regression analysis).

	Crude			Adjusted *		
	OR	95% CI	*p*	OR	95% CI	*p*
MetPB (Ref. MetPB concentration ≤ median)	0.73	0.37–1.46	0.376	1.14	0.46–2.82	0.775
EthPB (Ref. EthPB concentration ≤ LOD)	1.22	0.55–2.68	0.619	2.63	0.88–7.84	0.082
PropPB (Ref. PropPB concentration ≤ LOD)	0.49	0.24–1.01	0.054	1.45	0.44–4.71	0.540
Paraben total (Ref. Paraben total concentration ≤ median)	0.77	0.39–1.52	0.455	1.06	0.40–2.82	0.903

Abbreviations: Ref, Reference category; OR, Odds ratio; 95% IC: Confidence interval. * All analytes were adjusted for age, gender, and creatinine. MetPB was also adjusted for energy intake, EthPB for marital status, PropPB for energy intake, level of education, smoking habits, and marital status, and Paraben total for energy intake and level of education.

**Table 5:** Parabens level in saliva as influencing factors on overweight/obesity (logistic regression analysis).

		Crude	Adjusted *			
	OR	95% CI	*p*	OR	95% CI	*p*
MetPB (Ref. MetPB concentration ≤ median)	0.90	0.37–2.23	0.826	0.61	0.21–1.76	0.359
EthPB (Ref. EthPB concentration ≤ median)	0.45	0.18–1.12	0.087	0.41	0.15–1.12	0.082
PropPB (Ref. PropPB concentration ≤ median)	0.85	0.35–2.10	0.729	0.94	0.37–2.40	0.892
Paraben total (Ref. Paraben total concentration ≤ median)	1.14	0.48–2.74	0.764	1.00	0.38–2.62	0.995

Abbreviations: Ref, Reference category; OR, Odds ratio; 95% IC: Confidence interval. * All analytes were adjusted for age and gender. MetPB was also adjusted for energy intake and level of education, EthPB for physical activity and marital status, PropPB for marital status, and Paraben total for energy intake.

**Table 6:** Consulted literature which relates BMI to parabens in different biological matrices.

Reference	Matrix	N	Age	Country	Tendency/Relationship with BMI			
					MethPB	EthPB	PropPB	ButPB
Xu et al., 2022 [28]	Urine	300	2–60	China	-	Inverse	-	-
Lee et al., 2021 [24]	Urine	3782	19–86	Korea	-	Direct		
Feizabadi et al., 2020 [26]	Urine	178	21–>50	Iran	Inverse *	-	*	-
Hajizadeh et al., 2020 [50]	Urine	95 (w)	34.2 (m)	Iran	-	Inverse	-	-
Jala et al., 2022 [49]	Urine	52	18–31	India	-	-	-	-
Li et al., 2019 [23]	Urine	696	NR	China	-	-	Direct	-
Guo et al., 2017 [20]	Urine	436	3	China		Direct	-	-
Kang et al., 2016 [22]	Urine	2541	3–69	Korea	Direct *	-	Direct *	-
Berger et al., 2021 [21]	Urine	309	5	USA	-	-	Direct	-
Quirós-Alcalá et al., 2018 [25]	Urine	1324	6–19	USA	Inverse	-	-	-
Deierlein et al., 2017 [51]	Urine	1017 (w)	6–8	USA	-	-	-	
Kim and Chevrier, 2020 [27]	Urine	2564	3–79	Canada	-	Inverse (woman)	-	-
Güil-Oumrait et al., 2022 [29]	Urine	1015	11 (m)	Spain	-	-	-	-
Artacho-Cordón et al., 2018 [54]	Adipose tissue	144	+16	Spain	-	-	-	-
van der Meer et al., 2017 [52]	Brain	25	74 (m)	Netherlands	-	ND	ND	ND
Reimann et al., 2021 [53]	Placenta	229	30 (m)	Belgium	-	Direct	-	-

Abbreviations: NR, Not reported; ND, Not detected; (m), mean; (w), women; *, correlated parabens in matrices.

## Data Availability

Not applicable.

## Supplementary Materials

The following supporting information can be downloaded at: https://www.mdpi.com/article/10.3390/nu15051154/s1, Table S1: Comparison between control and cases included/not included subjects.
