# Supplementary material for: Presence of Parabens in Different Children Biological Matrices and Its Relationship with Body Mass Index
Source: Nutrients. 2023 Feb 24;15(5):1154. doi: 10.3390/nu15051154 (PMC10005709; doi:10.3390/nu15051154)
Supplement: Supplementary file 1 [file nutrients-15-01154-s001.zip › nutrients-2209676-supplementary.pdf]

Table S1. Comparison between controls and cases included/not included subjects.

		Cases (n=94)			Controls (n=137)		
		**Included (n=59)**	**Not included (n=35)**	***p***	**Included (n=101)**	**Not included (n=36)**	***p***
Gender (%)	Male	58.3	48.6	0.398 [a]	49.5	50.0	0.999 [a]
	Female	41.7	51.4		50.5	50.0	
Age, categorized (%)	≤ 10 yrs	75.0	57.1	0.108 [a]	79.2	88.6	0.312 [a]
	> 10 yrs	25.0	42.9		20.8	11.4	
Weight, kg	Median	53.30	51.20	0.758 [b]	25.45	26.60	0.813 [b]
	IQR	21.90	25.70		12.58	10.13	
Height, cm	Mean	140.37	141.67	0.682 [c]	127.79	126.05	0.641 [c]
	SD	12.93	17.81		20.68	13.60	
Energy Intake, kcal day^{-1}	Mean	2001.08	1900.93	0.409 [c]	2011.94	1925.31	0.574 [c]
	SD	452.98	441.29		512.39	345.10	
Physical Activity (out-of-school) (%)	No	38.6	44.8	0.646 [a]	41.4	38.1	0.999 [a]
	Yes	61.4	55.2		58.6	61.9	
Parents' level of education (%)	Primary	7.0	0		1.1	0	
	Secondary	52.6	41.4	0.141 [a]	24.7	23.8	0.882 [a]
	University	40.4	58.6		74.2	76.2	
Smoking among parents (%)	No	81.7	73.5	0.434 [a]	78.6	86.1	0.461 [a]
	Yes	18.3	26.5		21.4	13.9	
Marital status parents (%)	Married	77.2	65.5		92.1	76.2	
	Divorcee	17.5	31.0	0.354 [a]	3.4	23.8	0.004 [a]
	Single	5.3	3.4		4.5	0	
Urinary creatinine, g L^{-1}	Median	0.90	-	-	0.87	-	-
	IQR	0.77	-		0.60	-	

IQR: interquartile range; SD: standard deviation; *p*-Values < 0.05 are highlighted in bold; [a] Chi-square test; [b] U Mann-Whitney test; [c]Student´s *t*-test
